# Entropic Tension in Crowded Membranes

**DOI:** 10.1371/journal.pcbi.1002431

**Published:** 2012-03-15

**Authors:** Martin Lindén, Pierre Sens, Rob Phillips

**Affiliations:** 1 Department of Applied Physics, California Institute of Technology, Pasadena, California, United States of America; 2 Laboratoire de Physico-Chimie Théorique, CNRS/UMR 7083, ESPCI, Paris, France; 3 Division of Biology, California Institute of Technology, Pasadena, California, United States of America; University of Illinois, United States of America

## Abstract

Unlike their model membrane counterparts, biological membranes are richly decorated with a heterogeneous assembly of membrane proteins. These proteins are so tightly packed that their excluded area interactions can alter the free energy landscape controlling the conformational transitions suffered by such proteins. For membrane channels, this effect can alter the critical membrane tension at which they undergo a transition from a closed to an open state, and therefore influence protein function *in vivo*. Despite their obvious importance, crowding phenomena in membranes are much less well studied than in the cytoplasm. Using statistical mechanics results for hard disk liquids, we show that crowding induces an entropic tension in the membrane, which influences transitions that alter the projected area and circumference of a membrane protein. As a specific case study in this effect, we consider the impact of crowding on the gating properties of bacterial mechanosensitive membrane channels, which are thought to confer osmoprotection when these cells are subjected to osmotic shock. We find that crowding can alter the gating energies by more than 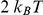 in physiological conditions, a substantial fraction of the total gating energies in some cases. Given the ubiquity of membrane crowding, the nonspecific nature of excluded volume interactions, and the fact that the function of many membrane proteins involve significant conformational changes, this specific case study highlights a general aspect in the function of membrane proteins.

## Author Summary

Biological membranes are a complex array of lipids and proteins. The typical bacterial membrane is made up of hundreds of copies of different species of membrane proteins embedded in a sea of different types of lipids. One of the distinguishing features of biological matter is the high degree of “crowding” to which the different macromolecules are subjected. In this work, we explore the consequences of such crowding in the membrane setting, building upon earlier work which has primarily focused on how crowding affects properties in the cytoplasm. The particular case study considered here centers on a class of membrane channels which respond to tension in the cell membrane serving to provide osmoprotection to cells subjected to osmotic shock. We explore how the critical tension at which these channels open depends upon the concentration of other membrane proteins, and conclude that it can be significantly higher at physiological protein densities compared to the intrinsic value measured in protein free membranes.

## Introduction

Cell membranes are packed full of proteins. The essence of various membrane inventories is that biological membranes are at least as much protein as they are lipid. Experiments on the occupancy of biological membranes by lipids and their protein partners provide a useful basis for making estimates of the possible consequences of membrane crowding. The presence of such high areal fractions of protein means that there is the possibility that the “crowding” effect can alter the free energies of different membrane protein conformations and the dynamics of the changes between these conformations as well. Indeed, over the last several decades, the importance of crowding effects in general has become a theme of increasing concern in physical biology [1]–[6].

The question of how the behavior of membrane proteins is altered by crowding effects has been explored much less thoroughly than their bulk counterparts [6]–[11]. As a concrete example of the way crowding might play out in membranes, we consider transmembrane proteins that have several conformations with different areal footprint. One particularly fascinating class of proteins of this variety are the mechanosensitive membrane channels. These proteins are thought to serve as safety valves for cells that are exposed to osmotic stress, opening up in response to increased membrane tension for the purpose of equilibrating the cells with their external environment [12]–[15].

To see how crowding might serve as an additional factor in the overall gating free energy balance for mechanosensitive channels, we consider the gating tension associated with the mechanosensitive channel of large conductance (MscL). Upon opening, at membrane tensions larger than 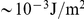, this channel undergoes a change in radius from roughly 2.4 nm to 3.5 nm [16]–[19]. As a result of this increased size, there is a reduction in the free area available for the surrounding membrane proteins resulting in an entropic driving force to keep the channel closed. The work presented here explores the relative importance of this effect compared to other contributions to the overall free energy budget for mechanosensitive channel gating.

In the remainder of this paper, we first examine various estimates of the degree of crowding in biological membranes. We then go on to explore the consequences of such gating for the free energy of the crowded proteins within the membrane, and the accompanying changes of the channel's gating tension.

## Results

### The degree of crowding in membranes

As a prerequisite to characterizing the functional consequences of membrane crowding, we must first estimate the extent of crowding found in different types of membranes. There are various ways to arrive at numerical estimates of the extent of crowding of membrane proteins in biological membranes. One key measurable quantity that reflects the fraction of membrane area occupied by proteins is the protein to lipid mass ratio which typically falls in the range 1–2.5 [20]–[23]. Assuming that transmembrane (TM) domains make up about half of the membrane protein mass [24] and have roughly the same density as the lipids results in the estimate that 30–55% of the membrane area in the bilayer plane is occupied by proteins. Sowers and Hackenbrock [25] obtained electron microscopy images of mitochondrial inner membranes after application of a strong electric field that made all proteins drift to one end of the membrane surface, and found that the packed proteins in those images occupy 40–50% of the total area. Ryan *et al.* [26] fitted a statistical mechanics model of steric exclusion to the distribution of fluorescently labeled membrane proteins on rat basophilic leukemia cells subject to an electric field, and extracted an area coverage of 55–75%. Direct experimental estimates of the protein area fraction in red blood cell plasma membrane and synaptic vesicles have yielded area fractions of 20–25% [21], [22]. In an extreme case, atomic force microscopy images of the photosynthetic membranes of *Rhodospirillum photometricum* cells [27] under various growth conditions show almost close-packed photosynthetic proteins arranged with nearly crystalline order. All of these examples tell the same fundamental story: membrane proteins are in very close proximity.

Another way of characterizing this crowding is by appealing to the number density which gives the number of membrane proteins per unit area of membrane. Aldea *et al.* [28] report that the five major outer membrane proteins (by mass) in *Salmonella typhimurium* have a total surface density of about 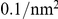 in a wide range of growth conditions. Neidhardt *et al.* [29] (p. 41) quote lipoproteins as the most abundant protein (by number) in *Escherichia coli*, with 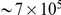 copies in the outer membrane of a typical cell. Estimating the area of a typical *E. coli* to be 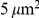 [30], this gives a density of about 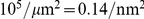. Another way to estimate a protein density is to consider the fraction of the genome that codes for membrane proteins. In *E. coli*, about 1/3 of the 4200 genes encode membrane proteins, and the total number of proteins is about 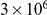 per cell [30]. If 1/3 of all proteins are evenly distributed in the two membranes, each membrane has about 500,000 proteins, or about 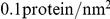. The areal and number densities estimated above are roughly consistent. If one assumes a footprint of 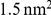 per transmembrane helix [21]–[23], and 3 transmembrane helices per protein (see below), a number density of 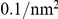 corresponds to an area fraction of 0.45.

There are other ways to think about the extent of membrane crowding, each with its own assumptions and merits, but regardless of these details the message will be the same. Biological membranes are crowded! For the purposes of this article, what these numbers tell us is that the mean spacing between proteins (estimated by evaluating 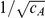) is only slightly larger than the proteins themselves, so that a significant fraction of the membrane area is occupied by proteins.

**Figure 1:**
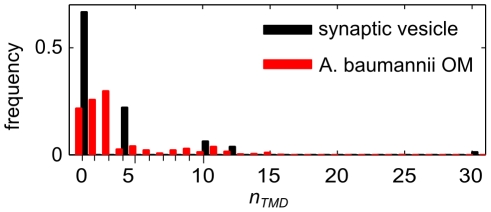
Relative abundance of membrane protein subunits with different number of transmembrane (TM) helices. The histograms are based on data for synaptic vesicles [21], and the outer membrane (OM) of the Gram-negative bacterium *A. baumannii* [33]. Proteins with no predicted TM domains were excluded.

Membrane proteins are not only abundant, they are also very heterogeneous, and vary significantly in size and shape [31]. Quantitative data on this heterogeneity is harder to come by, and we will therefore use the number of transmembrane helices 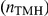 as an approximate indicator. Bioinformatic predictions of transmembrane regions [32] are routinely reported in surveys of proteins or putative protein-coding DNA regions [21], [24], [33], and range from one to several tens per protein subunit. Figure 1 illustrates two transmembrane helix distributions, based on a synaptic vesicle model [21] and a proteomics study of the outer membrane of the Gram-negative bacterium *Acinetobacter baumannii* [33], respectively. The latter is an average of three different techniques to estimate relative abundance, which differ significantly in specific cases, but lead to similar overall distributions (not shown). It is interesting to note the similarities in distributions in figure 1, both being dominated by proteins with a few TM helices, and spanning about one order of magnitude. However, there are several significant sources of uncertainty. For example, not all membrane proteins were detected [21], [33], and we have not accounted for aggregation of protein subunits into larger complexes.

In our calculations below, we will model membrane proteins by circular disks, and will need to estimate 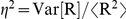, where 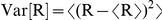 denotes the variance of 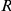. This quantity, which measures the variability of the projected protein area, enters into the more sophisticated treatments of the crowding effect discussed later in the paper. A useful approximation is 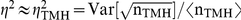, which comes from setting 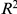 proportional to 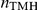. Excluding proteins with no predicted transmembrane domains, the synaptic vesicle and *A. baumannii* outer membrane protein data sets in figure 1 give 0.25 and 0.14 for 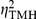, and 3.0 and 3.5 for the mean number of transmembrane helices, respectively. As we will see, these numbers indicate that size variability does not make a large quantitative contribution to the crowding effect, despite the quite broad distributions shown in figure 1.

### Crowding effects on gating

In light of estimated membrane protein crowding, our aim is to explore the implications of such crowding for channel gating. The total free energy change upon gating, 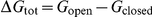, can be thought of as arising from multiple contributions. In particular, we have
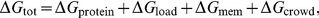
(1)where the first term reflects the free energy change associated with the protein degrees of freedom and their internal structural rearrangements, the second term refers to the potential energy of the loading device, and the third term characterizes the free energy of protein-lipid interactions, including the deformed membrane surrounding the protein that has been implicated as a key player in the gating of mechanosensitive channels [34]–[36]. The last term is the crowding-induced term. A membrane protein with a large cytosolic domain can potentially be crowded both by molecules in the cytoplasm, and by other membrane proteins. While the former effect has in fact been observed in the mechanosensitive channel MscS [37], it is the latter effect that forms the main substance of this paper.

The main conceptual point of the remainder of the paper can be stated simply as the idea that when the channel opens and changes its radius from “small” to “large”, there will be a free energy cost for the surrounding membrane proteins which we will refer to as crowders. In particular, these crowders will have their entropy reduced, which amounts to an effective pressure on the channel walls opposing its opening. To explore this claim, we will work in two distinct ensembles.

**Figure 2:**
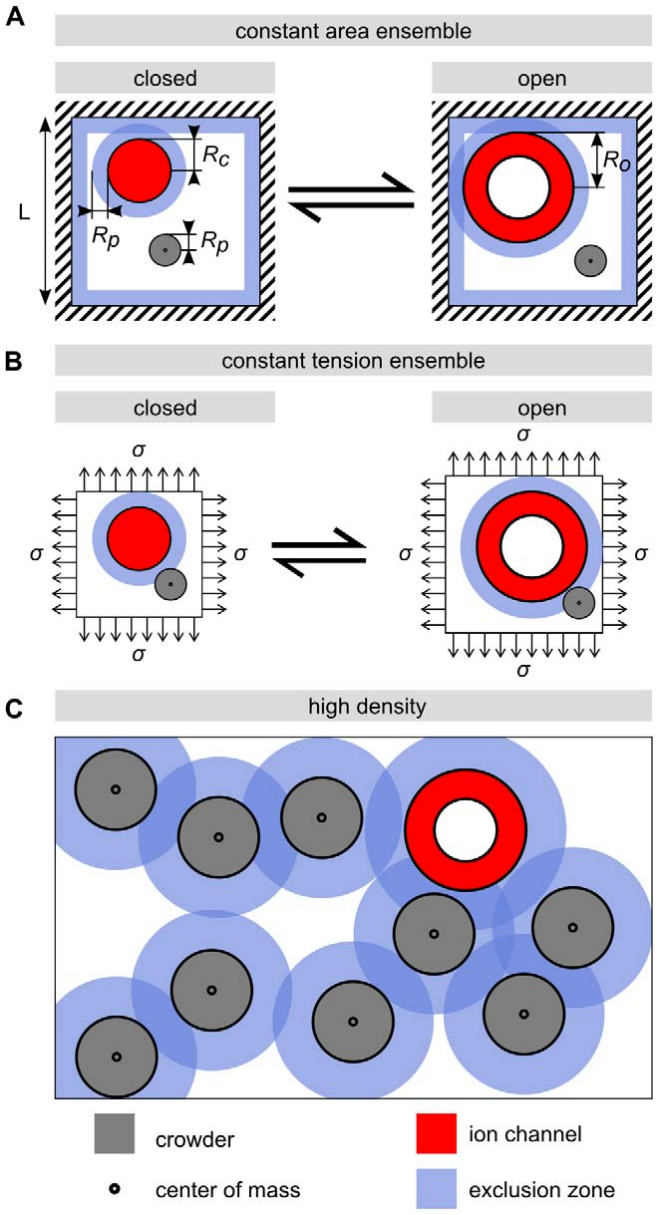
Excluded-area interactions and channel gating. (A) Gating of a channel (red) crowded by a single crowder (gray) of radius 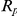 in the constant area ensemble, where the total surface area is fixed by the outer walls (dashed). (B) In the constant tension ensemble with applied tension 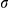, the total area increases as the channel opens, so that the total lipid area is conserved. For disk-shaped particles of finite size, the free area available for each center of mass is limited by the minimum distance between two centers of mass. This effect can be illustrated by exclusion zones of width 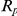 around each protein. In the constant tension ensemble, the reduced area for the crowders is due to larger exclusion zone in the open compare to the closed state. In the high density regime (C), the exclusion zones overlap, which complicates the analysis. We use scaled-particle theory to analyze this case.

In the (mathematically) simpler case, we imagine a two-dimensional membrane “box” like that shown in figure 2A, such that the overall area is fixed. When the channel goes from the closed to the open state, there is a net reduction in the available area for the remaining crowders, which results in an entropic tension that favors the closed state. We make no reference to the elastic cost of squishing the lipids to access this state, since it can be shown that this energy is negligible in comparison with our main contribution of interest which is the entropic effect (see supporting text S1, Sec. 1).

The second scenario imagines a loading device that subjects the membrane to some fixed tension on its perimeter, much like the springs that hold a trampoline under its state of tension. It can be shown that in this case, when the channel goes from the closed to the open state, the areal strain, and hence the lipid area available to the crowders, do not change significantly (see supporting text S1, Sec. 2). However, because of the change in the circumference of the protein, the exclusion annulus around the channel, indicated in figure 2, will be enlarged. Hence, there will still be an entropic tension which favors the closed state. In both cases, we make the implicit assumption that the number of lipids in the membrane does not change on the time scale of protein conformational changes.

To explore these two scenarios, we begin with the box of fixed area and use the simplest “ideal gas” physics to evaluate the change in entropy due to the loss of translational degrees of freedom when the channel goes from the closed to the open state. In particular, the translational entropy of one crowder can be computed as the logarithm of the area available to its center of mass,
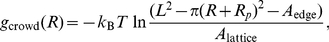
(2)where 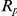 is the radius of the crowder, 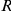 is the radius of the channel, 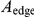 is the band of thickness 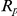 around the edge of the box from which the crowder center of mass is excluded (see figure 2 A). The denominator 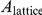 refers to a discretization length scale used in a lattice model for the entropy [30]. Hence, the numerator is the effective area available to the crowder in the 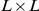 membrane patch, recognizing that the minimal center-of-mass distance between the crowder and channel is 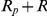 (see figure 2).

This expression can be simplified by expanding in the small parameter 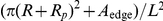. If we exploit this simplification and add the contributions from 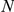 crowders, the difference in free energy between the open 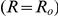 and closed 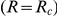 states due to the crowding contributions can be written as 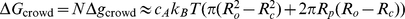, with 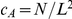 being the crowder concentration. These two terms have simple and intuitive interpretations that are serviced by noting that we can rewrite the area and circumference change, respectively, as 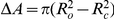 and 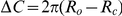. We can then divide the entropic crowding tension into a surface and a line tension, and write
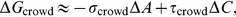
(3)in the constant area ensemble. In our “ideal gas” approximation, the surface tension is 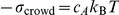, the familiar ideal gas law. The line tension, 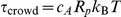, originates in the fact that the annulus of exclusion shown in figure 2 changes size upon gating. This contribution vanishes in the limit that the size of the crowders goes to zero. For both terms, we will need to appeal to our earlier estimates of protein areal concentrations to set the scale of the effect.

We can now consider the second scenario in which there is a fixed applied tension 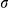, shown in figure 2B. Neglecting edge effects, the lipid area in which the crowders wiggle around does not change in this case, but the annulus of exclusion does, and hence the contribution of the entropy change to the free energy is given by the 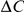 term only, i.e.,
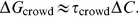
(4)At the same time, there is a relaxation in the energy of the loading device which takes the form 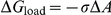 in the constant tension ensemble.

The treatment given above provides the simplest estimate of the crowding effect. However, as shown in figure 2C, things become more complicated in the high concentration limit. In particular, the amount of available area is much less than is suggested by the simple estimate above, where we made no reference to the way the crowders interact with each other. Neglecting these interactions underestimates the crowding effects. For 50% protein area coverage, the more accurate computations described below give increased surface and line tension terms by a factor of four and two, respectively. Note that the entropic effect increases in a highly non-linear fashion with the crowder area fraction, effectively diverging as one reaches the closed-packing limit. The effect we describe can thus be potentially much larger than the already substantial estimates of 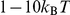 summarized below.

One way to think about this, illustrated in figure 2C, is in terms of exclusion zones around each crowder, analogous to the physics described by the van der Waals theory of gases. In the highly crowded regime, the theoretical difficulty is to compute the total size of the exclusion zones in a way that avoids double counting areas where multiple exclusion zones overlap. We use scaled-particle theory for mixtures of hard disks, an approximate equation of state that combines reasonable accuracy with analytical tractability [38]–[40], and has been widely applied to describe the effects of crowding [1], [2], [6]–[8], [11], [41]. The central results, for circular crowders, are presented in table 1, in terms of the concentration 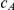, areal fraction 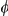, and, for non-uniform crowder size, relative size variance 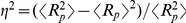. Details of the derivations are presented in the Models section below. The crowding-induced changes in gating energy still take the form of Eqs. (3) and (4), with only the line tension contributing in the constant tension ensemble. The more exact scaled particle theory gives larger crowding tensions.

**Table 1:** Entropic surface and line tensions induced by crowders.

	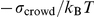	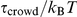
ideal gas	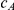	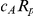
SPT, uniform crowders	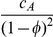	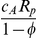
SPT, non-uniform crowders	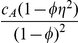	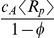

Entropic surface and line tensions induced by crowders, estimated by an ideal gas calculation and scaled-particle theory (SPT, see Eqs. (20) and (27)). The results are derived for the case in which a single circular protein increases its radius from 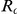 to 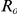 in the presence of circular crowders with radius 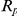. The non-uniform crowders case contains averages 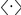 over the crowder radius distribution, and this size variation 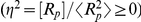 leads to a smaller surface tension effect compared to uniform crowders with the same mean size.

With these analytical results in hand, we now turn to the question of the actual magnitude of the crowding effect. To be concrete, we consider the case in which we have a membrane where the area is half lipids and half proteins (i.e. 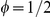), big enough to make the ideal gas estimates questionable. We consider a radius change of a single channel from 2.4 to 3.5 nm (as is appropriate for MscL [18]), and a crowder radius of 1 nm. For simplicity, we also neglect size variability and set 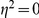 since using our estimate of 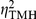 in the range 0.15–0.25 would only give a 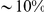 correction. This leads to an estimated crowder density of 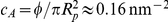. Using these numbers in the context of table 1, we get 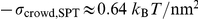, and 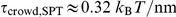. This translates to crowding-induced changes in total gating energies of 15.2 and 2.2 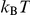, for the constant area and constant tension ensembles, respectively (see table 2). Even in the case of constant tension, most relevant to MscL, the crowding effect can have a sizable impact on channel gating.

**Table 2:** Estimated crowding effects on MscL gating.

	constant area		constant tension		
	IG	SPT	IG	SPT	units
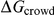	4.3	15.2	1.1	2.2	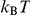
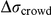	0.21	0.74	0.05	0.11	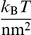

Different metrics for the effect of crowding on the gating behavior of a mechanosensitive channel. The first row shows the approximate changes in gating energies. The second row shows the corresponding increase in gating tension, 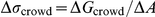, which can be measured directly in patch-clamp experiments. For comparison, the typical gating tension for isolated MscL is 0.3–1.3 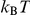/nm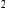. For MscL gating, the constant tension ensemble is the more appropriate model.

## Models

In this section, we review some basic results of scaled-particle theory, and derive the main results of the previous section and table 1. Motivations for some of the approximations we use, such as neglecting the area compression of the lipid bilayer, are given in the supporting text S1. See table 3 for a summary of the notation and symbols.

**Table 3:** List of symbols.

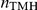	number of transmembrane helices	
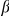	inverse temperature scale	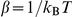
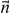	copy number vector	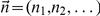
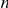	total copy number	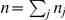
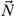	copy number vector for the crowders	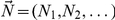
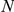	total number of crowders	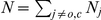
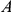	area	
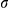	surface tension (2D analog of negative pressure)	
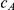	areal number density	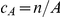
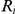	In-plane radius of species 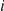 . In particular, 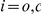 for the open and closed channel conformations respectively.	
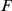	Helmholtz free energy (constant area ensemble)	
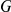	Gibbs free energy (constant tension ensemble)	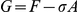
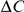	circumference change of channel	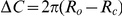
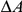	area change of channel	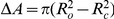
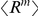	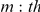 moment of the protein radius distribution	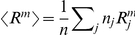
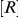	radius variance	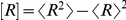
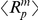	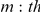 moment of the crowder radius distribution	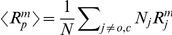
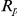	crowder radius, for the case of uniform crowder size	
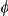	area fraction of disks or proteins	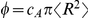
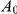	total area occupied by unstretched lipids	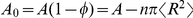
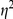	relative protein radius variance	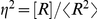
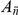	unstretched equilibrium area in constant tension ensemble	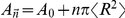 .

### Basic results of scaled particle theory

In this section, we restate some basic results of scaled-particle theory that serve as the basis for our calculations. We will express the results in terms of area, temperature, and particle copy numbers, since this is what we will use as thermodynamic control variables, but also quote the results in terms of variables like concentration 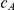 and area fraction 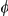 (see table 3).

Scaled-particle theory has been generalized to heterogeneous mixtures of convex particles [42]–[44], but we restrict our attention to mixtures of circular disks [40]. We will simply quote the results we need, and refer to the literature for details on the derivations [6], [38]–[40], [45].

We start with the canonical partition function for a collection of hard disks with radii 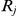 and copy numbers 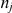, enclosed in an area 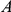. The crowding effect we are interested in comes from the configurational entropy of the proteins, and we therefore omit velocities and internal degrees of freedom, and neglect boundary effects. The remaining configurational partition function depends on the many-particle interaction energy, 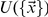,
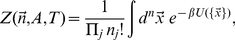
(5)where 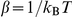 is the inverse temperature, 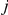 is the disk species index, and we use vector notation 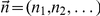 to denote the copy number distribution, with 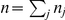 being the total number of disks (see also table 3). We next factor 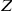 by multiplying and dividing by 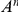, and write
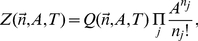
(6)where 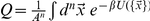 describes the deviation from ideal gas behavior due to the interaction energy 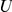, which we take to be simple hard-disk repulsion. (By construction, 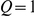 for an ideal gas, where 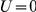 for all configurations.)

For the computations below, we will break down configurational changes as removals and insertions of particles of different sizes, and also consider area changes as a result of changed particle size. We will therefore need the chemical potential and surface tension of the disk mixture that is our protein model.

Scaled particle theory offers a simple equation of state that relates the surface tension (2D analog of negative pressure) exerted by the disks to the area footprint, number density, and size variation of the disks. Rewriting for example Eq. (6.7) of ref. [40] in our notation, we get
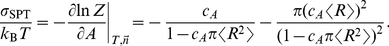
(7)(Note that we use the sign convention 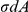 for surface tension-area work, which is the opposite sign compared to the pressure-volume convention 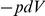 used in the original derivations of scaled particle theory). After substituting 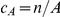, this expression can be brought to the more compact form
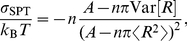
(8)where 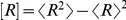 is the disk radius variance. Note how the size variability decreases the (negative) pressure through the variance term.

This can again be rewritten in terms of concentration, area fraction, and relative size variability by combining the concentration 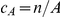 and the area fraction relation 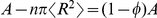, and then the relation 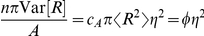, resulting in
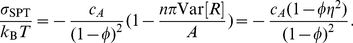
(9)When we consider area changes in the next section, the area integral of the surface tension at constant particle numbers will also come in handy, and we therefore integrate Eq. (8), and obtain
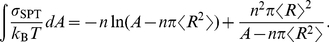
(10)


Finally, we will need the chemical potential. This is commonly divided into an ideal gas part plus a correction, called the excess chemical potential. Using 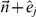 to denote the state with an added particle of species 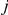, the excess chemical potential is defined as the ratio
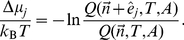
(11)Using manipulations similar to those that lead to Eqs. (7) and (8), the scaled-particle theory approximation given by, e.g., refs. [6], [40], can be rewritten in the form
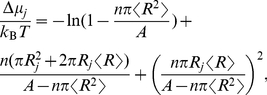
(12)where the averages should be computed with copy numbers 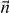, i.e., without the test particle present.

From the definition of 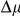 (Eq. (11)), one can see that 
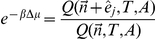
 also has a probabilistic interpretation, namely as the probability that a test particle can be inserted somewhere in the fluid without overlapping with the other particles. This observation, which is exact, is in fact the starting point for one way to derive scale-particle theory (see e.g., [6], [40]), by using a clever approximation to account for the overlapping exclusion zones in figure 2 C.

Finally, the chemical potential is given by the ratio of partition functions,
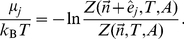
(13)If we substitute Eq. (6) and then Eq. (12), we get the scaled-particle approximation to the chemical potential, namely,
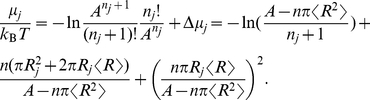
(14)


### Gating transition in the constant area ensemble

With the results of the previous section in hand, we are now in a position to derive our main results. Specifically, we will consider a situation with a single channel crowded by other proteins that do not change their configuration. We denote the copy number vector and total number of these crowders by 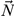 and 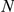 respectively. The state with a channel in state 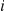 (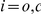 for the open and closed state respectively) will then have the copy number vector 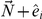.

In the results and discussion sections, we use 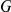 to denote a generic free energy. In the following derivations, we will be more precise, and use 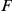 and 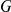 for the free energy in the constant area and constant tension ensembles, respectively. In the thermodynamic limit, they are related by a Legendre transformation 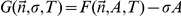, where 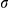 is the surface tension.

When computing the gating energy changes, we expand in various small parameters. Specifically, we will consider the total area, or total lipid area, to be much larger than the area of a single protein of any species, but comparable to the total crowder footprint 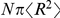. This means that 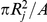 is a small parameter for all protein radii 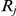, but 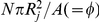 is not small. In a typical *E. coli* cell, 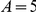 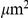, which means that 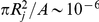 (for 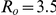 nm). We will neglect such small terms.

To compute the free energy changes of a conformational change at constant total area, e.g., changing a particle from species 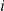 (say, a closed channel) to species 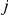 (say, an open channel), we subdivide the reaction into one insertion and one removal, by multiplying and dividing by the partition function of the intermediate state,
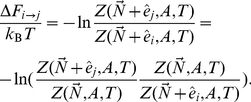
(15)Splitting the product of ratios, we can compare with Eq. (14) to identify the free energy change as the difference of chemical potentials for the two configurations,
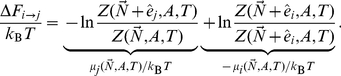
(16)This means that we can use Eq. (14) with 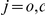 to compute the entropic contribution to the free energy change. Using 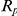 to denote crowder radii, we get
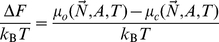
(17)

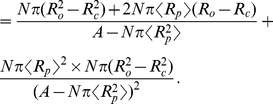
(18)To simplify, we first identify changes in area 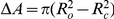, and circumference 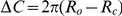,
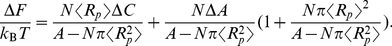
(19)Next, we use the same simplifications that lead to Eq. (9), and end up with
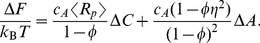
(20)The coefficients of 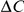 and 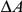 are the line and surface tensions tabulated on line three of table 1. The negative surface tension of the crowders (Eq. (9)) acts to oppose an increased radius of the protein, because increasing the protein footprint decreases the area available to the rest of crowders. The quantities in these coefficients should be computed without the channel present (although computing them with the channel present would only make a small difference). The properties of the channel itself only enter through 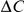 and 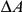. We obtain the uniform crowders result (line 2 of table 1) as a special case, by replacing the mean radius by a single value, 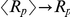, and set the coefficient of variation, 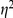, to zero.

Next, we consider the constant tension ensemble, and show that we recover only the line tension effect, i.e., the 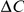 term, in that case.

### Gating transition in the constant tension ensemble

For the constant tension ensemble, the statistical mechanics recipe is to introduce an external tension 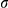, i.e., an external loading device with energy 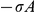. We also include a term 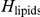 for lipid elastic energy as a function of area, and integrate over all areas,
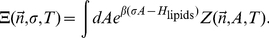
(21)As we show in the supporting text S1, real membranes are too stiff for changes and fluctuations in lipid area to give significant contributions to the gating energy of a single channel. This means that the above integral will be dominated by the area 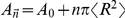, where 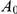 is the total unstretched lipid area. To good approximation, we can therefore set 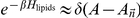, and think of the lipids as having constant area and infinite stiffness. This makes it easy to evaluate the area integral,
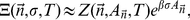
(22)and we recover the free energy of the constant tension ensemble as the Legendre transformation of the free energy for the constant area ensemble,
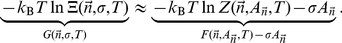
(23)


We now return to our test problem, and again denote the crowder copy numbers by 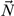, the presence of a channel in state 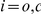 by 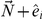 etc. We can then divide the total free energy change into three contributions: removal of a closed channel at area 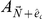, an overall area change 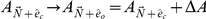 with no channel present, and insertion of an open channel at area 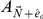:
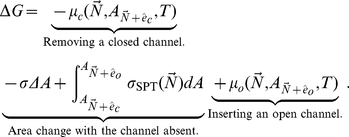
(24)Substituting Eq. (10) and Eq. (14), and assuming that the crowder background does not contain any other channels 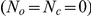, we get (after collecting terms)
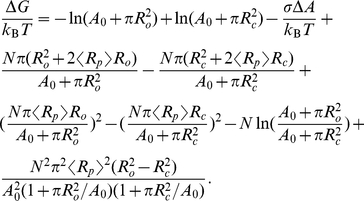
(25)Next, we Taylor expand in the small parameters 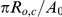, collect coefficients of 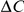 and 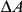 (most of which cancel), and end up with the following lowest order result:
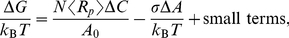
(26)Noting that 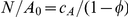 and discarding the small terms, we finally get
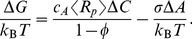
(27)Comparing with the constant area result of Eq. (20), we see that the contribution from the crowding surface tension has canceled, but that the coefficient of 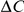 is the same, namely the line tension in table 1. The extra term 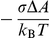 reflects the work done by the loading device during the area change, and is independent of crowding conditions.

## Discussion

Membrane proteins in cellular membranes are crowded. Estimates based on data from a broad range of organisms and experimental techniques [20]–[28] indicate that membrane proteins occupy area fractions ranging from 20% to well over 50% in different cell membranes. Crowding induces an entropic tension in the membrane, which favors membrane protein conformations with smaller areal footprint and circumference. This effect can be understood qualitatively using simple free area arguments, but quantitatively meaningful estimates require more sophisticated theories. We have used scaled-particle theory for hard disk mixtures [6]–[8], [38]–[40], [45] to compute the crowding induced line and surface tensions (see Models, Eqs. (20) and (27), and table 1). As a case study, we apply these results to estimate the influence of crowding on the gating tension of the bacterial mechanosensitive channel MscL. This channel is thought to act as a safety valve for cells under osmotic stress, opening up in response to high membrane tension in order to avoid membrane rupture [12]–[15].

**Figure 3:**
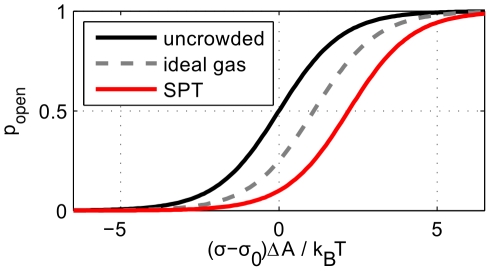
The effect of crowding on the open probability as a function of applied tension 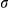
 **.** The graphs illustrate the ideal gas (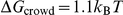) and scaled-particle theory (SPT, 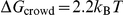) results of table 2, using the constant tension ensemble as is appropriate for MscL. All non-crowding contributions to the gating free energy are lumped together in the gating tension 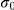.

There are different ways to quantify the influence of crowding on gating behavior (see table 2). One way to present the significance of our results is by appealing directly to the curves that provide the probability of channel opening as a function of the driving force. For the case of a “two-state” channel, which transitions back and forth between distinct closed and open states, the open probability is 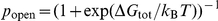, where 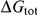 is the energy difference between the closed and open states, and depends upon the driving force (such as tension, voltage or ligand concentration). In our case, the driving force is the tension, and we can rewrite 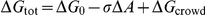. The first term corresponds to all contributions of Eq. (1) that do not depend explicitly on crowding or applied tension. We can rewrite it in the simpler form 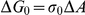. Figure 3 shows the gating probability 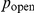 as a function of 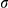 both for a single isolated channel and for the case in which crowders are present.

An alternative way to decide if the effect is big or small, is to compare it to some reference energy (or tension). The first relevant energy scale for comparison is the thermal energy 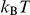, the energy scale in the Boltzmann weight 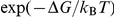 in the open probability above. Our numerical examples in table 2 all change the gating free energy by 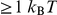. A second relevant energy scale is that associated with the gating of various mechanosensitive channels. The gating properties of channels such as MscL have been measured using several different species of lipid molecules in the surrounding membrane. The outcome of these elegant experiments is that the gating energies have typical values of 5–20 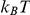 [17], [46] and corresponding gating tensions in the range of 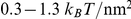. In the presence of spontaneous curvature inducing lipids, these energies and tensions are even smaller (or even negative, meaning that the channel opens spontaneously without any applied tension) [17]. The change in gating tension due to crowding is 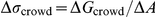, and we get numbers in the range 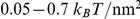.

The entropic cost of channel opening in a crowded solution of membrane proteins has so far been discussed only with reference to hard core repulsion between proteins. It is however well known that membrane-mediated interactions may emerge from the overlap of the membrane deformations surrounding neighboring proteins, such as those arising from a thickness mismatch between the hydrophobic protein core and the membrane average thickness[34], [35], or a non-cylindrical shape of the transmembrane region [34], [35], [47]. Beside the hydrophobic mismatch itself, the strength, and even the sign of such interactions depend on many factors, including membrane stiffness to bending and stretching, and the monolayer's spontaneous curvature. The range of these interactions is comparable to the protein size itself, and hence could be expected to influence the effect of crowding on the gating energy significantly.

**Figure 4:**
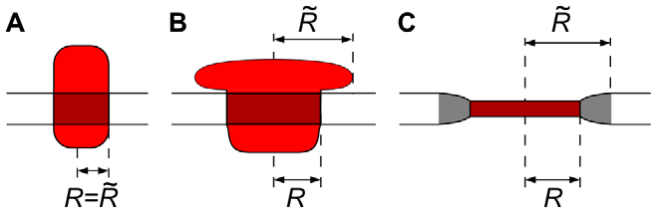
Mechanisms for different excluded area for proteins and lipids. One difference between the hard disk model of membrane proteins (A), and more complex protein structures (B,C) might be thought of in terms of different effective radii 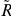 and 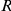 for steric exclusion of surrounding proteins and lipids respectively. A protein (red) with a large domain outside of the bilayer (B) might exclude surrounding proteins, but not lipids, from approaching the transmembrane domain (dark red). Similarly, proteins with different hydrophobic thickness than the surrounding bilayer (C) generate a local zone of deformed lipid bilayer (gray) that effectively excludes other well-matched proteins. Horizontal lines indicate the surrounding lipid bilayer.

The rich and interesting many body effects that can emerge from local membrane deformations are outside the scope of this paper. However, our calculations offers some qualitative insight into the sensitivity of the crowding effect to structural features of the involved proteins, which also includes some effects of hydrophobic mismatch. In our hard disk calculations under constant tension, the entropic surface tension cancels from the gating energy contribution (between Eqs. (3) and (4), and in Eq. (25)), when the increase in channel area is balanced by an increased total area. This cancellation reflects an underlying assumption in the disk model of membrane proteins, which effectively models membrane proteins as cylinders (figure 4A), from which lipids and other proteins experience the same area exclusion.

Real membrane proteins, however, can have complex shapes that violate this assumption [31], for example due to large domains outside the bilayer that do not directly affect the local ordering of the lipids, but provide additional steric interaction with other membrane proteins, as sketched in figure 4B. Many membrane bound receptors that bind bulky ligands near the membrane surface [48]–[51], yield complexes with a similar shape. We would expect significant crowding effects on both the binding kinetics and the stability of the complex for these systems, similar to what has been seen for surface adsoption [9]–[11], [41]. There are also examples of membrane proteins whose bulky cytoplasmic domains undergo substantial conformational changes, such as the mechanosensitive channel MscS [37], [52] and the 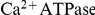 [53]. Hydrophobic mismatch might play a similar role in a surrounding of mostly well-matched proteins (figure 4C).

The presence of conformations with such structural features might remove the surface tension cancellations, and thereby change the dependence of gating energy on crowding in a qualitative way. One should thus consider the two ensembles studied here (constant area and constant tension) as limiting cases capturing the range of phenomenon that can be expected for real membrane proteins. Trying to imagine more quantitative estimates of these effects points towards new and interesting questions, both theoretically and regarding structural features of whole membrane proteomes. For example, it seems likely that the large cytoplasmic domain of MscS experiences a different crowding environment than it's transmembrane part. First, a large cytoplasmic domain can be crowded by macromolecules in solution [37]. Second, it can only interact directly with those membrane proteins that also possess bulky cytoplasmic domains, not with those that mainly consists of transmembrane helices. Finally, the MscS transmembrane part might be shielded from direct interaction with the transmembrane parts of other proteins with bulky cytoplasmic domains, if those domains are large enough.

The present analysis has as its key outcome the hypothesis that under sufficiently crowded conditions, membrane proteins can influence each others conformational changes through an entropic tension. Though we explored the consequences of that idea for one particular channel, given the great diversity of membrane proteins and the high degree of crowding in many membrane types, we expect that such effects could be common.

## Supporting Information

Text S1 Additional calculations to justify some assumptions mentioned in the main text.
(TEX)
